# Researching Human-Cattle Interaction on Rangelands: Challenges and Potential Solutions

**DOI:** 10.3390/ani11030725

**Published:** 2021-03-07

**Authors:** Maggie Creamer, Kristina Horback

**Affiliations:** Animal Behavior and Cognition Laboratory, Department of Animal Science, University of California-Davis, One Shields Ave, Davis, CA 95616, USA; mlcreamer@ucdavis.edu

**Keywords:** animal personality, animal welfare, beef cattle, human-animal interaction, rangeland

## Abstract

**Simple Summary:**

Research investigating the influence of human-animal interactions on beef cattle production and welfare often occurs during stress-inducing contexts, such as restraint and transport. Given that beef cattle which graze on expansive rangelands do not frequently interact with humans, assessment of animal response to humans may be inaccurate if they are only recorded during such stressful context. Future research aimed at identifying the causes and impacts of individual human–cattle relationships may consider conducting experiments while the cattle are in a low stress context, such as while ruminating, resting, or, grazing. By adapting methods used to research wildlife response to humans, researchers may be able to better understand how animal personality and previous experience can influence cattle production and welfare.

**Abstract:**

Human-animal interaction (HAI) research spans across many scientific fields and animal taxa. For livestock species, HAI research tends to focus on animals that are managed in close proximity with humans such as poultry, dairy cattle, and swine. Given the nature of rangeland cattle production, HAI research with beef cattle often occurs in and around the processing environment. This high arousal context may skew behavioral and physiological responses by the animals due to the potentially negative interaction. The aim of this review is to describe cattle production on rangelands, examine the considerations and limitations of current HAI research used to evaluate interaction quality or traits of rangeland cattle, identify contexts in which rangeland cattle interact with humans, and provide recommendations for improving future HAI research with rangeland cattle. Current research delineating individual differences in response to humans by beef cattle occur during routine husbandry and management on rangelands (pragmatic) and in a research context (experimental). Human-cattle interactions can be distinguished based on the quality and goal of the interaction into four broad categories: human presence, human approach, human contact, and restraint. Limitations of HAI research with rangeland cattle are identified and reconciled by recommendations for HAI research that can take place outside of the processing environment (i.e., while cattle are ruminating, resting or grazing on rangelands).

## 1. Introduction

Human-animal interaction (HAI) research spans a wide range of scientific fields, such as psychology, animal science, zoology, and veterinary science, and animal taxa, such as companion, zoo, laboratory, wildlife, and agricultural animals [1]. HAIs can be defined as mutual behaviors that arise in the human and the animal that are based on mutual perceptions [2,3]. The objectives, methods, and outcomes of human-animal interaction research can vary depending on the scientific field and the taxa of non-human animals. In the field of psychology, HAI research is primarily focused on how bonds between humans and companion animals can improve human health, provide therapy, and alter child development [4]. Thus, these studies typically measure the outcome of human-animal interactions, bonds, and relationships as they pertain to the human rather than the animal [1,5,6,7]. HAI research with agricultural animals, however, focuses more on the impact that humans may have on animal well-being and welfare [1,3,8,9,10]. This is reflected in the disproportionate use of terminology: human-animal interaction or relationship is used when agricultural animals are the subjects, while human-animal bond is used when companion animals are the subjects [1].

The quality of an interaction between humans (e.g., stockpeople) and livestock can be influenced by current sensory stimulation (auditory, visual, olfactory and/or tactile) and previous associative learning paradigms [3,11]. For example, lambs appeared to establish stronger social bonds with humans that provided positive tactile contact (e.g., petting) than with those that provided no physical contact [12]. Pigs which learned to associate humans with a positive reward (e.g., feed) also approached humans more often and demonstrated a reduced fearful response towards humans [13]. Human-livestock interactions are important to consider in animal management for several reasons, including: (i) these interactions have been found to impact behavior, performance, and welfare of the animal [11,14,15] and (ii) these interactions play an important role in behavior and welfare research of these animals in terms of understanding their fear and stress response [3,16,17,18,19,20]. There are also more human-centric reasons to study human-livestock interactions such as evaluating the well-being of stockpeople and how that affects handling as well as examining the ethics of livestock care [21].

The positive or negative impacts of human-livestock interactions are often studied through the animal’s behavioral and physiological responses to handling or restraint [3,11]. The magnitude and type of behavioral or physiological response [22] to handling can provide information about what an animal perceives as stressful, painful, or generally aversive. An animal’s behavioral and physiological response to handling can also provide important insight into how animals vary in behavioral type (i.e., temperament, personality, coping style [17,23,24]). Although the terminology with which to identify consistent variation in behavior when animals are confronted with human interaction varies according to research field and topic [25], consistent animal personality has been found to relate to health and immunity, production traits, foraging behavior, breeding success, maternal behavior, affective state, and cognition. For example, cows that were classified as having a consistent ”fearful” temperament in restraint tests also produced calves with lower birth weight and lower average daily gains than those that were classified as having a ”calm” temperament [26]. In fact, research has shown that the quantity and quality of handling style with livestock can impact the growth rate, pregnancy rate, and characteristics of meat, wool, egg or milk quality and yield among animals of various personalities [27,28,29,30,31,32,33]; reviewed by [3].

The majority of the livestock HAI literature is focused on species which are raised and managed in close proximity to humans, such as dairy cattle, swine, and poultry, and cover a variety of issues related to approach, contact, and the quality of the HAI [11,34,35]. In contrast, most HAI research involving beef cattle and sheep breeds occurs in or around the contexts of processing, transport, and slaughter (i.e., working facilities [15,16,23,36,37,38,39]) even though there are other contexts in which humans interact with cattle and sheep such as herding, maintenance of fences or water, supplement provision. This trend towards studying human-animal interactions when the animals are most accessible (i.e., during transport) could produce biased data given that the animals are responding more toward the high-arousal context, rather than the human itself. It is understandable why most research conducted with rangeland beef cattle occurs in or around the processing context not only from a practical standpoint, but also a logical standpoint depending on the goal of the research. If the goal of the research is to examine animal responses to specific high-arousal handling techniques that might present challenges to bovine welfare, then human-animal interaction research in and around processing is the most relevant context in which to conduct this type of research. However, if it is to evaluate a cow’s innate response to humans or to evaluate individual traits of cattle, other contexts outside of the processing environment in addition to those within the processing environment can give a more holistic representation of cattle responses to humans or traits of individuals.

Given this concern, the objectives of this review are to (i) describe cattle production on rangelands to provide a comprehensive background for HAI in this population of animals (ii) examine the considerations and limitations of HAI research to evaluate interaction quality or traits of rangeland beef cattle (iii) identify the contexts in which rangeland beef cattle interact with humans, and (iv) provide recommendations for improving human-animal interaction research with rangeland beef cattle.

## 2. Beef Production on Rangelands

Rangelands are defined as expansive land areas, which are not covered in forest or ice, and are not cultivated or built upon [40]. Globally, rangelands host a variety of diverse species of flora and fauna and provide ecosystem services, such as carbon sequestration, nutrient cycling, provision of high-quality habitats, pollination, and soil fertility [41,42,43]. Both domesticated ruminants and wild ruminants can be found grazing across global rangelands, contributing to nutrient cycling by fertilizing soil, reducing fuel-loads for prevention of wildfires, and maintaining vegetation health and habitat diversity [44,45,46,47,48]. The fundamental difference in grazing behavior between wild and domestic ruminants is the level of human intervention and control. One of the most common domestic ruminants to be managed on rangelands, cattle (*Bos taurus* and *Bos indicus*), originate from the pivotal region of human agriculture, the Fertile Crescent region of the Near and Middle East [49,50]. The wild ancestor of cattle, the auroch (*Bos primigenius*), possessed specific characteristics which predisposed them for domestication, including gregarious social structures and polygamous mating systems. Selective breeding practices over thousands of years have resulted in the desirable behavioral characteristics for domestic cattle, including reduced flight zones (e.g., minimal distance from humans which results in movement by animal) and reduced general aggression [51]. Although humans have managed to select these traits over thousands of years to make animals safer to handle, measures are taken to ensure that there still exists a robust flight zone to allow easier herding by human, vehicle, dog, or horse approach.

Global beef cattle production has historically relied on the ability of cattle to consume native vegetation types as a high-protein diet source [52] that are inedible for humans and on land that is difficult to cultivate or build upon. From this standard, the extent to which cattle production has been modified throughout history depends on the geographical location. Some countries and regions have realized relatively marginal change in how cattle are managed and continue to rely entirely on pasture grazing and forage finishing (e.g., New Zealand, Australia, South Africa and subregions of South America and Europe; [53,54,55]), while other countries, like the U.S. and Brazil, have transformed largely to rely on partial pasture grazing followed by the “feeding stage” in a dry- or feedlot. This method of geographically differentiating grazing and feeding stages may continue to spread globally as the human population grows and encroaches upon grazing land [41,52,53,56]. Ultimately, local beef production differs depending on the environment, feed and forage type and availability, how forage is managed, the cattle breeds used, and the value chain and market.

Rangelands and pasture provide the optimal environment on which cattle in the U.S. can raise offspring until they are old enough to be transported to feedlots or dry-lots and fed a diet of concentrated grain for fattening [53,57]. Depending on the region of production, mature cattle will calve either in the fall or the spring [58]. Sexually mature female cattle, or dams, retained for breeding purposes will stay with their young until they are weaned around 6–8 months depending on the ranch or farm, within which time the dam will be bred again to continue the gestation cycle for the following year [58,59]. Thus, dams can give birth to calves at the same time every year and have a gestation period of over 9 months (281–286 days [60]). Dams typically remain in the herd until they are no longer financially viable as a result of age-associated issues like diminished fertility (may require multiple breeding) or fecundity (do not have a calf or have relatively small calves), calving issues (still born, abortion, require assistance), or health problems [58,59]. Mature cattle herds grazing on expansive rangelands are typically minimally managed by ranchers because of the effort to find, transport, and process them at central facilities that are located away from grazing allotments.

## 3. Considerations and Limitations of HAI Research in Rangeland Cattle

There are several notable oversights in human-animal interaction research conducted with rangeland beef cattle because of the unique situation posed by animals that are infrequently handled and living on expansive landscapes. Neutral or positive human-animal relationships can develop over time when frequent interactions are possible [3,61,62,63], especially because animals can become habituated to handling, and thus, display a lower fear response [64,65,66]. Habituation is a learned reduction of a behavioral response following repeated exposure to a stimulus [67]. Due to the fact that the infrequent handling of rangeland beef cattle typically occurs during painful or mildly aversive procedures, this livestock population may be less likely to habituate to handling or develop positive or neutral human-animal relationships (e.g., [68], demonstrated in pigs).

**Figure 1:**
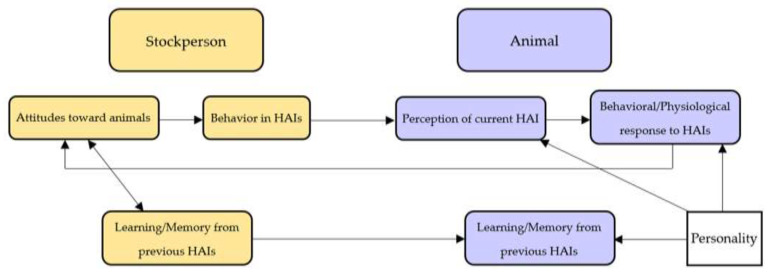
Factors which can influence the behavioral and physiological changes experienced by the animal subject(s) during human-animal interaction (adapted from Hemsworth, 2003 [11]).

These infrequent, aversive handling procedures also occur almost exclusively in the processing environment (chute, corrals, processing barns and facilities), thus range cattle can begin to associate these locations with fear and negative emotions and are able to anticipate procedures based on environmental cues ([3,19,62] Figure 1; Table 1). Human-animal interaction research that occurs in and around these locations are already operating within a context of fear and stress to which rangeland cattle have not been habituated. This phenomenon, known as conditioned place aversion, is a combination of classical conditioning and associative learning. It describes when environmental cues become associated with negative events after repeated exposure to the pairing of the cue and event resulting in an animal’s tendency to avoid locations and environmental cues that were previously paired with the negative experiences [69]. Cattle have exhibited sufficient memory capabilities to learn aversive handlers and locations of aversive handling procedures, especially given the salience of fearful situations [19,62,70,71,72,73]. Grandin et al. (1994) [74] demonstrates that it is difficult for cattle to unpair negative experiences in a given location despite other experiences that may follow, which is especially notable on rangelands where there are infrequent handling opportunities. In addition to memory of past experiences causing fear and stress in processing environments [71,72,75,76,77], immediate procedures surrounding human-animal interaction research may also impact outcome measures. For example, if a study is using chute score and flight speed to measure fear of restraint by humans during or after some aversive or painful experience (e.g., branding), it would be difficult to disentangle fear of handling from expression of pain or anxiety caused by the procedure itself or from the sounds and stress hormones of other cattle that have been processed prior to the focal cow [78,79,80,81]. In animal welfare literature, there are often associations and predictions made between handling procedures and welfare, advising alterations to handling procedures (tone of voice, tools used, etc.) [19,37,71,82,83] or to facility structure and context that would benefit animal’s welfare state whilst they are handled [14,37,83]. However, if it is difficult to disentangle innate fear of humans and basic human handling procedures from fear of the environment or context, then advising alterations to human and/or environmental factors may be misinformed and conflated.

Meaningful comparisons between individual range cattle (e.g., personality or temperament) is also difficult given the numerous differences in quality and quantity of human interaction experienced by cattle. If human-animal interactions are utilized to make assumptions about underlying personality traits in cattle, researchers must consider the interdependence of personality traits and past experiences in the processing environment. Personality in non-human animals is typically defined as individual differences in behavior that are consistent over time and across contexts [84]. If consistently “flightier” cows receive rougher handling in the chute, they may show a heightened fear response in the chute context compared to conspecifics not receiving this handling quality [3,62,85]. Fear responses of cattle in the processing environment will reflect previous experience and memory, due to conditioning of cues, conflated with an animal’s ability to cope with fear (related to individual personality). This situation presents challenges for assessing coping ability relative to other animals since not all animals receive the same prior treatment (Figure 1). This is especially true when measures of the effort of stockpeople are used to make indirect assumptions about animal’s intrinsic traits or their response to handling [3,39,82]. For example, Hemsworth et al. (2011) [39] quantifies the frequency at which the animal receives tactile, auditory or visual interaction from the human such as touching, slapping, clapping, talking, or whistling.

Gregarious livestock species also display a stress or fear response to social isolation [3,22,85,86,87,88,89,90]. This response can differ between species, but also between individuals within species because of differences in their personality (i.e., sociality dimension; [22,84,91]; Figure 1). Conducting human-animal interaction research with cattle undergoing social isolation that may differ in their response to social isolation makes it difficult to attribute behavioral responses to fear of human interaction or to fear of isolation without clear controls. When human-animal interaction approaches are used for insight into differences in animal personalities, researchers must adopt a multidimensional framework for animal personality [22,91]. Multidimensional approaches to personality that involve animal reactions to several different situations typically with controls, gradients of exposure, or biological or physiological validation reveal a comprehensive understanding of animal personality [22,91]. By validating measures across dimensions, behaviors can be deduced as arising from differences between individuals rather than differences in behavior attributed to some environmental factor. Due to individual animal personality and its influence on animal behavior, researchers should consider individual differences in personality, coping style, and temperament when evaluating responses to handling even if that is not the direct goal of the project because this may explain some variation among or between animals that cannot be explained by the handling procedure itself [16,17,23]. In general, a multidimensional approach to understanding animal personality can diminish the potential for welfare concerns caused by near-sighted herd management decisions. Temperament, a related yet distinct quantifiable subset of traits [25], is used by ranchers in some cases to make breeding and selection decisions for herd maintenance [92]. However, one-dimensional temperament assessments that take place solely in the processing enviroment may not reflect the complex, personality dimension of the focal animal [22,25,91]. Breeding and selection decisions based on temperament can negatively alter herd dynamics and cattle welfare if, for example, heavier, calm-temperament cows (evaluated by chute score) are kept in the herd because they pose less of a threat to worker safety while also having higher production value, however these same individuals on range may display limited protective behavior of their calves from predators [26,92]. Calmer, heavier cows may also suffer more severe health issues since energy is diverted to maintaining their larger body-weight rather than other biological functions like immunity (e.g., resource allocation theory [22,92,93,94]. Further, if animals are reacting to social isolation or fear of the context caused by memory of rough handling, ranchers may be making incorrect judgements of animal personality and breeding and selection decisions for herd management would be misinformed.

### 3.1. HAI in Rangeland Beef Cattle

Human-cattle interactions on rangelands can occur in two contexts: pragmatic and experimental. Pragmatic HAIs occur during routine husbandry, management, and processing of rangeland beef cattle. Experimental HAIs occur during specific research procedures designed to identify animal response in an empirical or standardized setting. HAI research involves observations and assessments with either or both pragmatic and experimental contexts of HAIs. A selected summary of the current literature on HAI in beef cattle is provided in Table 1. The inclusion criteria for literature to be included in this table includes: beef (not dairy) cattle as the focal species, behavioral observations recorded, and, keywords such as “human-animal interaction”, and “cattle”. Within rangeland beef production, both pragmatic and experimental human-animal interactions can be categorized based on the focus being human presence, human approach, human contact, or restraint by human (or by chute with human present). There are many similarities and differences in frequency and nature of human-animal interactions when they occur via routine management and when they occur in an experimental, standardized context for research on beef cattle behavior and welfare. Pragmatic HAIs and their juxtaposition against experimental HAIs are not as thoroughly reviewed as experimental HAIs in the literature [3,8,16,17,23] although this is an especially important distinction in rangeland beef cattle. Thus, the following sections seek to highlight lacking details about pragmatic rangeland beef cattle HAIs while giving a thorough overview of both pragmatic and experimental HAIs that occur with rangeland beef cattle, describing what they entail and what measures can be taken from them, categorized by human presence, approach, contact, and restraint.

#### 3.1.1. Human-Presence

Human presence entails auditory, visual, or olfactory interaction or, more commonly, some combination of the three. Pragmatic human presence with rangeland beef cattle mostly occurs in conjunction with approach, restraint, and physical contact, however there may be a few instances that human presence occurs without an additional level of human intervention. Pragmatic human presence on rangelands occurs when ranchers are monitoring or observing cattle, using a specific type of herding, or assessing water, supplement, or fencing. Experimental human presence in research typically involves a human standing in front of the chute or in an open arena (often a corral or pen in processing environment) with an individual or group of cattle (Table 1). Often the human is unfamiliar to cattle (to mitigate the confound of past experiences with a familiar human) and assumes a standard position for a specified amount of time across tests, for example sitting on a stool with still hands (e.g., [95,96]). The behaviors recorded in experimental human presence tests include latency to approach the human, average proximity to the human, and intentional physical contact made with the human (e.g., [95]). Pragmatic and experimental interactions can be classified as positive, neutral, or negative depending on the context in which the interaction occurs, past experience with the interacting human, and the behavioral type of the individual experiencing the interaction, which all contributes to how an individual cow perceives the interaction [35,36,97]. For example, a familiar, gentle, handler delivering supplement or cleaning/providing water in a trough is likely a positive pragmatic interaction because it is paired with a reward [19,36]. If herding occurs with a noise stimulus (e.g., horn “honk”) paired with a food reward (typically in the back of a vehicle), which the cows are urged to follow, this can be classified also as a pragmatic human presence event paired with a reward. Pragmatic human presence interactions might describe simple observations of cattle when the rancher is on horseback, in a vehicle, or on foot. In contrast to these common occurrences of familiar humans in proximity, human presence in an experimental context may create a more negative experience for cattle due to the novelty of the stimuli and/or the unfamiliar environment (Table 1). For example, a stationary researcher in an open corral with one cow may elicit stress-related behavioral and physiological responses in the animal given the novelty of the situation and the uncommon experience of social isolation. Pragmatic human presence may also elicit stress responses in the cattle depending on the context in which it occurs (unfamiliar or familiar human and/or situation, past experience with human and/or situation, social isolation).

#### 3.1.2. Human-Approach

Human-approach typically involves a combination of auditory, visual, and olfactory interaction between the focal cow and the human. Pragmatic human approach occurs during herding, calving, and sorting. During herding on rangelands, humans are typically approaching cattle on horseback or with a vehicle. Human approach in the calving setting refers to how the mother cow perceives the approaching human because she is typically not physically contacted by the human, unlike the calf. During calving, ranchers will approach the cow-calf pair and process the calf, which commonly involves weighing and vaccinations, but can include painful procedures like castration of males and branding depending on the ranch [98]. Sorting commonly occurs prior to breeding, processing, or transport and involves humans approaching cattle and using flight zones to guide their movement. Experimental human approach tests with rangeland beef cattle often occur in and around the processing infrastructure such as permanent or temporary corrals and pens (Table 1). Human approach tests can occur with or without conspecifics in close proximity to the focal beef cow in the corral and pen and involve a human approaching a focal cow (or group of cows) with a standard approach position and from a standard distance across repeated measures (Table 1). For example, as demonstrated in Freitas-de-Melo et al. (2019) [99], the same observer approached the animal from 10 m at a pace of 1 step/second without visual contact while the animal was alone in an open yard and while the animal was grazing in the paddock. Measures recorded during the human approach test include flight distance, the distance between the human and the cow at which the cow steps away from the approaching human, as well as body and ear postures. Pragmatic and experimental techniques likely vary in their degree of inducing stress responses depending on the handlers and the urgency of approach. Pragmatic low-stress handling and herding techniques have gained popularity amongst beef cattle ranchers because they not only may improve the welfare of their animals by reducing fear and stress responses, but also the ability to move or work cattle as well as production value [100,101,102,103]. Horseback herding versus herding using a vehicle likely invokes reduced fear reactions in cattle because vehicle herding occurs more rapidly and involves noises that may be startling for cattle. Calving and sorting are also potentially stressful instances of pragmatic human approach because the human represents a challenge or obstacle to the cow-calf bond. Experimental approach-tests likely vary in their degree of perceived stress to the cow based on if the cow is isolated, in an unfamiliar environment, and/or the characteristics of the approaching human (familiarity, size, gender, posture, urgency) [3].

#### 3.1.3. Human Contact

Human contact refers to physical contact made between the human and the animal and as such encompasses a combination of auditory, visual, olfactory, and tactile interaction with the focal cow. Pragmatic contact by humans can occur during the various management events previously mentioned. Physical contact, to some degree, may occur during sorting, processing, breeding (via artificial insemination [AI]), and calving. Contact during calving refers to the human making direct physical contact with the calf during processing. Procedures that occur when cattle are restrained at working facilities such as vaccinations, hoof-checks, pregnancy checks, in some cases breeding (if AI), branding and castration involve varying degrees of pragmatic human contact [81,98,104,105]. Sorting and moving cattle through working facilities may also require human contact with hands, flags, raddle paddles, electric prods, or other tools used to coerce cattle into moving. Herding is mostly conducted without direct physical contact, but on some ranches herding with tools used to contact and move cattle does occur. While experimental human contact tests are rare among beef cattle HAI literature, these studies typically involve a range in the quality and quantity of tactile contact, from gentle touch or stroke to the face or body, to slaps and electric prod use, depending on the goal of the experiment (Table 1). This body of literature also contains varied research objectives from conditioning animals to human touch [106,107] to looking at fear responses to this extreme degree of human proximity [108]. Thus, methods of experimental human contact and behaviors measured are not as homogeneous than other types of HAIs. Most studies measure behavioral and physiological responses to human contact in some capacity, for example body posture, respiration rate, escape attempts, and vocalization. Pragmatic and experimental human-contact is likely accompanied by heightened stress responses in rangeland cattle because physical contact occurs infrequently and is typically paired with aversive experience (prodding, transport, vaccinations, branding, pregnancy checks; [89,106]. Even gentle physical contact (stroking, petting) likely provokes a stress response in cows because of the novelty of the situation until cattle are habituated to the experience (stress responses evidenced by a necessary habituation period for contact [106] and increase in reactivity and basal heart rate to stroking [108]).

#### 3.1.4. Restraint

Restraint while a human is present is not always explicitly defined in cattle literature reviews and empirical studies because it encompasses studies involving cattle moving through a chute, cattle restrained in a small pen, and cattle manipulated by humans to remain in a small area (i.e., with either nothing, temporary fencing, flags, or ground marks). Pragmatic and experimental restraint in this review will therefore encompass when cows are restrained in the chute, restrained by a small pen (0.8 × 2.6 m [109]), and what is titled both the ‘docility test’ and/or ‘restraint test’ by several empirical studies in which cattle are restrained in a pen corner by a handler (2 m × 2 m [95,108,110,111,112], or unspecified size [113]). Restraint tests in the chute or a small pen involve a combination of auditory, visual, and olfactory interaction with the human while restraint tests in a pen corner involve the latter three modalities listed as well as occasional contact with an object the handler is using to restrain the cow (e.g., a stick [108,110,112,113]. Pragmatic restraint while a human is present occurs during processing procedures and transport. It commonly involves a working facility if ranchers have access to one. For processing (vet procedures, breeding, etc.), cattle are restrained in pens, corrals, and/or a working chute (hydraulic or manual) along with other infrastructure within the working facility (lead-ups, bud boxes, alleys). Pragmatic restraint might also include tethering cattle. Transporting cattle between allotments, feedlots, and abattoirs, can also be classified as pragmatic restraint. HAI research of cattle often includes measures of chute score and exit velocity, which are measured during and after restraint in a squeeze chute, respectively. Chute scores are assigned subjectively by raters observing animals live or through video recordings of behaviors in the chute. Typical chute score scales tend to vary between one and six, with one signifying a calm or docile behavioral profile and six signifying an aggressive, struggling behavioral profile (e.g., [114,115,116,117,118,119]). Exit velocity describes the amount of time it takes for a cow to travel a given distance (i.e., speed) after being restrained in the chute (e.g., [114,115,116,117,118,119]). Exit velocity, although not measured during restraint, is a common measure of the animal’s response to restraint and thus is included in this restraint section. These measures may be taken before, during, or after routine processing events (weighing, vaccinations), or they can occur outside of routine husbandry procedures (Table 1). Other objective measures might be collected for restraint behavior in the chute such as recording total number of movements (e.g., [120]), standard deviation of weight measurements over time (as a proxy for movement in the weigh scale by Bruno et al., 2016 [121]), and vocalizations on a continuous scale. Experimental restraint tests involve humans constraining animals to a certain location within a larger pen or arena (i.e., one corner of the pen) and measuring the ease at which the handler is able to perform this task. Ease of handling is measured by recording the amount of time it takes for cattle to be constrained, the amount of times the cow tries to exit the designated area, and the amount of time the cow is within the designated area (also known as the docility test;, e.g., [95,110,111]). Pragmatic restraint during processing and transport as well as experimental restraint is assumed to be acutely stressful, as evidenced by physiological, behavioral, and biochemical responses of cattle exposed to restraint, and is likely due to several factors such as social isolation, proximity to humans, and overwhelming sensory stimuli [15,34,36,79,122,123,124,125,126].

**Table 1:** This table displays examples of scientific literature examining human-animal interactions in beef cattle during routine husbandry and management on rangelands (pragmatic) and those utilized in a research context (experimental). The location of the experiment is where observation of interaction or an interaction manipulation takes place. Isolation from conspecifics indicates an attempt to physically isolate beef cattle from other group-mates.

Experiment Location	Isolated from Conspecifics	Context	Literature
**Human Presence**			
Pen/arena	Yes	Experimental	[95,96,108,112,113,119]
Pen	No	Experimental	[38]
Pasture/paddock	No	Experimental	[127]
Chute	Yes	Experimental	[95,108,120]
Chute/race	No	Pragmatic	[119]
Abattoir	No	Pragmatic	[39]
**Human Approach**			
Pen	No	Experimental	[117,127]
Pen	No	Pragmatic	[95,110]
Pen	Yes	Experimental	[95,99,113]
Home environment	No	Experimental	[99,107,109,128]
Feeding alley	No	Experimental	[106]
**Restraint**			
Chute	Yes	Pragmatic	[82,95,96,99,108,114,115,116,117,118,119,120,121,129,130,131,132]
Pen corner	Yes	Experimental	[95,108,110,111,112,113]
Small enclosure	Yes	Experimental	[109]
**Human Contact**			
Abattoir	No	Pragmatic	[39]
Chute	Yes	Pragmatic	[96,129,130]
Small enclosure	Yes	Experimental	[113]
Home environment	No	Experimental	[107]
Chute	Yes	Experimental	[108]

## 4. Recommendations for Future HAI Research in Rangeland Cattle

There is immense value in studying human-animal interactions in rangeland cattle because they can provide insight for animal welfare and production [3,11], as well as how individual differences in behavior manifest in altering landscapes and biodiversity of surrounding ecosystems [133,134,135,136]. However, this value is at risk of being undermined by methods that do not take into account the unique characteristics of range cattle outlined above. In order to address and mitigate these confounds, researchers should consider doing field experiments with rangeland cattle in settings outside of the processing area and in the presence of conspecifics.

### HAIs in Contexts Outside of the Processing Environment

**Figure 2:**
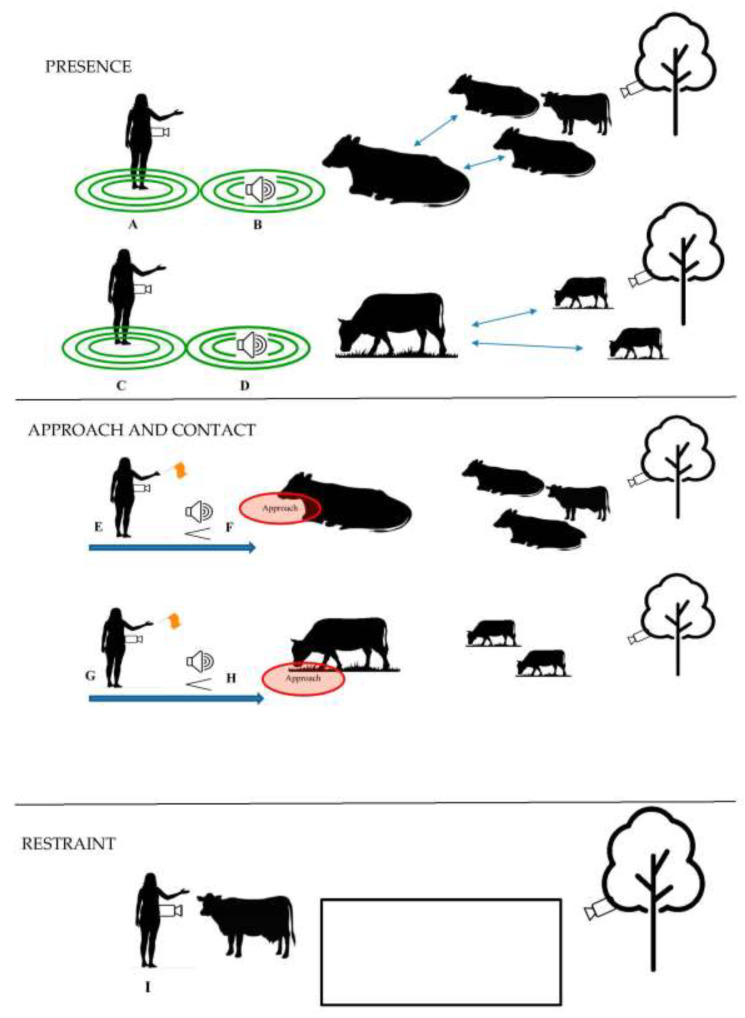
Schematic of potential HAI experiments to be conducted on-range with beef cows. Tests while cattle are resting (A, B, E & F) can occur near water and shade; tests while cattle are grazing (C, D, G & H) can occur along grazing routes. Resting sites and grazing routes can be determined by preliminary observations of cattle on rangeland. Based on the goals of the experiment, researchers will set a pre-determined distance from which the focal cow is separated from conspecifics depicted by arrows in schematics A–D (e.g., 1 m). Human presence testing is displayed in the top panel. An unfamiliar human (A & C) stands within 10 m of focal cow for 10 min while focal cow is resting or grazing. Hidden speakers (B & D) can be placed at central location where cattle rest or graze, and when focal cow is within 10 m of speaker, unfamiliar human voice can play for 10 min. Green concentric circles indicate proximity zones to measure focal cow’s approach/avoidance distance from human or speaker. Human approach and contact is displayed in the middle panel. An unfamiliar human (E & G) begins 10 m from focal cow and approaches focal cow diagonally from the left side of the focal cow’s head with hand extended and eyes averted at a steady pre-determined pace (e.g., one step per second). If experiment is to capture response to human contact, researcher can pursue physical contact with hand or inanimate, extended object (e.g., flag) in the same standardized manner. Hidden speaker (F & H) can be placed at central location where cattle rest or graze, when focal cow is within 10 m of speaker, unfamiliar human voice plays at ascending volume for 10 s to emulate approaching human. Restraint of cow by human is displayed in the bottom panel. An unfamiliar human (I) begins restraint attempt from 10 m while cow is resting or grazing and attempts to move cow into a square area (2 m × 2 m) marked by spray paint or temporary fencing. Multiple hidden cameras can be fastened onto the unfamiliar human in the test or trees nearby the testing area. Distance from focal cows at beginning of tests (in this example, 10 m) may need to be adjusted based on the herd flight zone sensitivity. Potential behaviors to record include vigilance duration and quality, body/ear position, vocalization quality, approach/avoidance or flight distance based on presence, approach and/or contact, and escape behavior. Limitations of such experiments include characteristics of human, quality of human approach, temporal effects (time, day, season), presence/behavior/identity of adjacent non-focal cows, and cow arousal level.

Depending on the specific goals of the human-animal interaction study, researchers should consider assessing human-animal interactions outside of the processing environment in order to be able to disentagle innate fear of humans or human handling from fear of context and/or to understand multidimensional personality traits of the animal. Extensive rangelands are defined as large swaths of land on which domestic and wild ruminant grazing takes place. Notably, this is a difficult setting to conduct human-animal interaction research because of the distribution of animals across terrain, traversing difficult terrain with equipment, and logistics of contacting or attempting to contact animals that have ample space to move away. However, the benefits of conducting such research arguably outweigh the costs (e.g., [137]). Schematic examples of human-interaction tests (presence, approach, contact, and restraint) that can be conducted with range cattle are presented in Figure 2. The schematic examples involving human presence and approach with an unfamiliar human are adapted from studies that assess human interactions in a ‘home’ or grazing environment [99,107,109,128], presence and approach with a speaker and restraint schematic examples are derived from experiences undergone by authors on unpublished pilot studies and studies with wildlife that utilize speakers.

Cattle confronted with human-interaction while resting or grazing on range are not responding within a spatial constraint, signifying that they can display a more comprehensive behavioral repertoire when confronted by human-animal interaction (i.e., longer flight distances, ability to hide, ability to rapidly move away; [3,137]). Additionally, this method does not involve previous and recent aversive experiences that might impact results of the study such as herding, capturing, handling, and transporting cows to the processing environment [3,138]. Human-animal interaction research is still meaningful and useful when conducted in and around the processing area, but this should be compared to human-animal interactions conducted in the field setting that are free of confounding variables like past experience, memory, and spatial constraint. Not only might this provide insight in how cattle perceive familiar or unfamiliar humans outside of a given context, but may also validate our ability to draw conclusions based on cattle responses to humans in processing environments.

In order to conduct such interaction research on widely distributed animals, researchers can take advantage of the behavior of cattle. For example, cattle grazing patterns have been found to consist of a period of rest and drinking water in the middle of the day bookended by grazing bouts in the early morning and evening [139,140]. Evening grazing bouts can extend throughout the night and flow into morning grazing bouts. Researchers studying human-animal interactions in beef cattle can take advantage of the resting and drinking period in the middle of the day to conduct interaction research as long as the watering sites are accessible. The reliance of animals on water and the ability of researchers to access watering sites within pastures or rangelands provides an opportunity for researchers to interact with animals in settings apart from the processing environment. Animals likely will be in the presence of conspecifics when these tests are conducted, which is arguably better for interpreting beef cattle behavior without the confound of their response to social isolation [22,86,141] and because they are resting and grazing around conspecifics in their daily lives [102,134,136].

Implementing standardized, generalizable tests with cattle which graze on expansive and heterogeneous rangelands is a formidable obstacle. However, creative solutions to avoid these issues are possible (Figure 2), and may be found in wildlife ecology literature (e.g., [142]). Rangeland pastures are quasi-natural for grazing ruminants in that these extensive landscapes are minimally stocked and cattle are able to traverse expansive areas of diverse flora and fauna. Innovative technologies, such as Global Positioning System (GPS) tracking devices, accelerometers, Radio-frequency identification (RFID), game or motion sensor cameras, and long-range speakers are often utilized by behavioral ecologists when investigating animals’ response to human encroachment, noise pollution, or other manmade events [143,144,145,146,147]. Many of these technologies output useful sensor data that allow researchers to gain a sense of animal movement patterns and behavior without interfering directly with the animal or to understand longer-term effects of human-interaction events. For instance, GPS tracking collars paired with accelerometers have been used in cattle to understand variations in grazing patterns across individuals, seasons, and years both on larger spatial scales (with GPS) and smaller local scales (e.g., bite rates and rumination via accelerometers) (e.g., [148,149,150,151,152]). Researchers can use this data in practical grazing management, for instance, to compare before and after human intervention of implementing management tools to achieve optimal cattle distribution such as placement of protein supplement and/or herding to specific locations on rangeland. Likewise, researchers could use these technologies in adapted field human-interaction assessments to understand longer-term variations in cattle behavioral responses. Researchers may also consider adaptations of less invasive assessments that can be performed on range landscapes, such as fecal-sampling of cattle after a human intervention event. The cattle industry estimates breeding values (EBVs) of individual cows based on heritable traits from the dam and sire, such as carcass quality, average daily gain, offspring weaning weight, and other performance parameters. Beef breeders have begun including certain personality traits in their breeding estimates, such as docility and ease of handling assessed most commonly by chute scores and exit velocities [92,153]. Accurate, unbiased behavioral and physiological data collected using HAI methods outside of the processing context may better inform behavior and personality-focused EBVs for cattle selection.

## 5. Summary & Conclusions

The goal of HAI research in beef cattle may include: (i) examine animal’s ability to cope with stressful or fearful situations (i.e., unveil coping style, temperament, personality, breed or age differences) (ii) determine behavioral and physiological indicators of fear and/or stress (iii) examine an animal’s response to specific humans and handling procedures. Research that fulfills these goals contribute to our knowledge of livestock behavior and welfare in numerous ways. Understanding under what circumstances animals undergo behavioral and physiological changes due to fear of human interaction and how to identify these key transitions help to inform best handling practices since animals cannot communicate directly with us. The ability to correctly classify animals as having certain personality types and coping styles enables humans to select animals that best suit their environment and/or adapt management practices to accommodate behavior types of animals [17]. Insight into why an animal behaves or responds in a certain way and being able to locate a pattern of responses allows beef cattle ranchers to be more dynamic, specialized, and proactive in improving animal welfare.

Given that rangeland beef cattle do not require daily care, HAI for this livestock population is infrequent and somewhat aversive. This sporadic contact with humans is intentional as it reduces human labor hours and allows for a robust flight zone to be maintained for herding purposes. A primary limitation of the HAI research with rangeland beef cattle is the unintentional bias in behavioral and physiological data collected in response to high-arousal contexts (e.g., herding, transport, restraint, vaccinations), rather than in response to humans alone. This bias in behavioral and physiological data could thwart ranchers’ abilities to understand cattle behavior and undermine subsequent attempts to improve cattle welfare through specialized husbandry and management decisions.
